# Extent of exposure to environmental tobacco smoke (ETS) and its dose-response relation to respiratory health among adults

**DOI:** 10.1186/1465-9921-6-13

**Published:** 2005-02-08

**Authors:** Wasim Maziak, Kenneth D Ward, Samer Rastam, Fawaz Mzayek, Thomas Eissenberg

**Affiliations:** 1 Syrian Center for Tobacco Studies, Aleppo, Syria; 2 Department of Health & Sport Sciences, and Center for Community Health, University of Memphis, Memphis, USA; 3 Department of Epidemiology, Tulane School of Public Health and Tropical Medicine, New Orleans, USA; 4 Department of Psychology, Virginia Commonwealth University, Richmond, USA; 5 Institute of Epidemiology & Social Medicine, University of Muenster, Muenster, Germany

## Abstract

**Background:**

There is a dearth of standardized studies examining exposure to environmental tobacco smoke (ETS) and its relationship to respiratory health among adults in developing countries.

**Methods:**

In 2004, the Syrian Center for Tobacco Studies (SCTS) conducted a population-based survey using stratified cluster sampling to look at issues related to environmental health of adults aged 18–65 years in Aleppo (2,500,000 inhabitants). Exposure to ETS was assessed from multiple self-reported indices combined into a composite score (maximum 22), while outcomes included both self-report (symptoms/diagnosis of asthma, bronchitis, and hay fever), and objective indices (spirometric assessment of FEV_1 _and FVC). Logistic and linear regression analyses were conducted to study the relation between ETS score and studied outcomes, whereby categorical (tertiles) and continuous scores were used respectively, to evaluate the association between ETS exposure and respiratory health, and explore the dose-response relationship of the association.

**Results:**

Of 2038 participants, 1118 were current non-smokers with breath CO levels ≤ 10 ppm (27.1% men, mean age 34.7 years) and were included in the current analysis. The vast majority of study participants were exposed to ETS, whereby only 3.6% had ETS score levels ≤ 2. In general, there was a significant dose-response pattern in the relationship of ETS score with symptoms of asthma, hay fever, and bronchitis, but not with diagnoses of these outcomes. The magnitude of the effect was in the range of twofold increases in the frequency of symptoms reported in the high exposure group compared to the low exposure group. Severity of specific respiratory problems, as indicated by frequency of symptoms and health care utilization for respiratory problems, was not associated with ETS exposure. Exposure to ETS was associated with impaired lung function, indicative of airflow limitation, among women only.

**Conclusions:**

This study provides evidence for the alarming extent of exposure to ETS among adult non-smokers in Syria, and its dose-response relationship with respiratory symptoms of infectious and non-infectious nature. It calls for concerted efforts to increase awareness of this public health problem and to enforce regulations aimed at protecting non-smokers.

## Introduction

The deleterious effects of exposure to ETS on the respiratory system of adults and children is well documented [1-4]. Exposure to ETS not only influences respiratory health among those affected but also leads to increased health care utilization and costs because of respiratory problems [5,6]. For example, a recent study investigating more than 10,000 children in Germany showed that the risk of emergency department visits and hospitalizations from asthma was more than double for children exposed to 10 or more cigarettes/day compared to less exposed children [6]. ETS exposure was found to be associated with respiratory symptoms, abnormal lung functions, and increased bronchial responsiveness in children and adults [1-4,7,8]. Of special significance for developing countries, lower respiratory infection, the single most important cause of death for children below the age of 5 years, has been found to be associated with exposure to ETS [1,9-12]. However, with most studies of ETS exposure and respiratory health being done in developed countries, local evidence to promote clean air policies and to enforce existing policies are lacking in most of the developing world.

The situation with exposure to ETS in developing countries is likely to be aggravated by the widespread of smoking, lack of restrictions regarding indoor smoking, overcrowded housing conditions, and failure of health services [13-15]. Cigarette smoking in Aleppo is affecting some 70% of men and 20% of women aged 30–45 years, with an average of 1.2 cigarette smoker per household [16]. Moreover, Syria as well as other countries in the Eastern Mediterranean region (EMR) are experiencing an alarming increase in the popularity of waterpipe smoking [17,18]. Although this form of smoking is generally considered an outdoor social practice, research done at the Syrian Center for Tobacco Studies (SCTS) shows that more serious smokers demonstrate a predominantly individual use pattern (home, and alone) [19]. As such, waterpipe smoking may be an important source of ETS due to its emissions and length of smoking bouts [18,20]. Assessment of exposure to ETS, therefore, needs to encompass all information relevant to the studied setting and the smoking patterns of the target population.

Despite these troubling facts, there is a dearth of research examining the relationship between ETS exposure and respiratory health in developing countries, with the few available studies limited by poor outcome definition, lack of systematic exposure assessment, and inadequate control of confounding [21,22]. A recent review of this subject identifies a major limitation of available data being restricted to industrialized nations [3]. Generally, the use of different methodologies and markers of exposure and outcome precludes arriving at a clear picture of the relationship between ETS exposure and respiratory health. For example, not all studies have found a relationship between exposure to ETS and lung function impairment [23,24], some did not find a dose-response relationship [25], while others demonstrated gender-specific effects [24,26]. The reliance on single or historic indicators of exposure (spouse's smoking, maternal smoking during pregnancy) can lead to an imprecise estimation of exposure or recall problems [27,28]. Previous quantitative and qualitative research done in Aleppo has identified ETS as a potentially major health hazard in the indoor environment [16,29]. The current study, which is the first to assess respiratory health of adults and its relation to ETS exposure in Syria, is based on a population-based household survey (Aleppo Household Survey, AHS) done in Aleppo in 2004 utilizing multiple self-reported indicators of exposure and outcome as well as expired breath CO and spirometry.

## Methods

### Population and sampling

**Figure 1:**
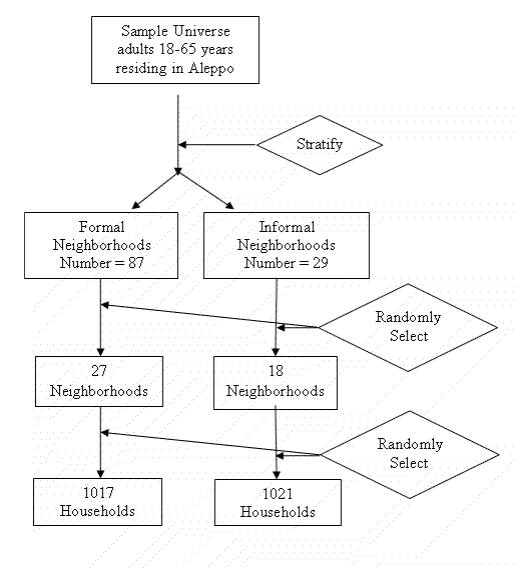
The overall sampling scheme of the Aleppo Household Survey. In the 1^st ^step the target population was divided into two strata, formal and informal zones (where residential areas are build illegally or on a land not designated for housing). In the next step residential neighborhoods were selected with PPS, and within selected neighborhoods households and one adult within each were selected with equal probability.

The target population consisted of adults 18–65 years of age residing in the greater city of Aleppo (around 2,500,000 inhabitants). Detailed description of the sampling design and procedures of the AHS is reported elsewhere and illustrated in Figure 1[16]. Briefly, stratified cluster sampling was used where residential neighborhoods of the city were stratified into two strata: formal and informal; according to the official description of the municipal registry (Figure 1). From each stratum, residential neighborhoods were randomly selected with probability proportional to size (PPS). Within each neighborhood, households were selected with equal probably and an adult was randomly selected from each.

The survey was conducted between May-August 2004, and the protocol and the informed consent documents were approved by the Institutional Review Boards at the University of Memphis and SCTS.

### Design and procedures

This interviewer-administered survey involved six, 2-person, mixed gender teams of surveyors equipped with notebook computers programmed to record questionnaire responses and measurements using a custom data entry program (Delphi programming language and SQL server DBMS). The questionnaire included 8 main sections; socio-demographics, general health and disability, chronic disease, respiratory health, household members' health, environmental health, smoking, and ETS exposure. For the assessment of respiratory health and risks, the questionnaire was developed based on relevant instruments (especially the European Community Respiratory Health Survey-ECRHS, the International Study of Asthma and Allergy in Children-ISAAC, and ETS exposure assessment instrument developed by Eisner and colleagues), as well surveys done in Syria [29-35]. Next, the survey instrument and strategy were modified in terms of format, content, language, response categories, and recall period based on formative work conducted with key informants involved in the provision of health care as well as with residents, in addition to piloting in 20 randomly selected households [16,29]. After being randomly selected, all study participants underwent the detailed study interview and objective measurements; height using a sliding wall meter (Seca, Germany), body weight using digital scales (Camry, China), expired breath carbon monoxide (CO) using breath CO monitors (Vitalograph, US), and lung function tests using portable spirometers (Micro-plus, UK) according to a standard protocol (16).

### Exposure

Data from self-reported non-smokers (both cigarettes and waterpipe), validated by breath CO levels ≤ 10 ppm (Table 1) [36,37], were analyzed for this report. ETS exposure assessment utilized responses to multiple inquiries about short- vs. long-term, indoor vs. outdoor, and cigarette vs. waterpipe exposures, as well as sensory irritation due to ETS exposure (a marker of intensity of exposure) (Table 2) [29-35]. Spouse's and parental smoking assessment included inquiries about length, intensity, and type of smoking (cigarette, waterpipe). Occupational exposure to respirable pollutants other than ETS was assessed by asking those involved in paid work whether they are exposed to dust, foams, smoke or other respirable particles at their work categorized as no exposure, mild exposure (a day or less weekly), moderate exposure (more than a day per week but not daily), and severe exposure (almost daily). Parental allergy was assessed by asking whether the respondent's parents ever suffered from respiratory or nose allergy with responses categorized into none, father, mother, or both.

**Table 1:** Basic indicators of Aleppo Household Survey (AHS) participants (n = 2038), and non-smokers satisfying criteria for inclusion in the analysis (n = 1118)

	All participants n (%)	Non-smokers with breath CO ≤10 ppm n (%)
Age		
18–29 years	736 (36.1)	450 (40.3)
30–45 years	874 (42.9)	418 (37.4)
46–65 years	428 (21.0)	250 (22.4)

Gender		
Men	921 (45.2)	303 (27.1)
Women	1117 (54.8)	815 (72.9)

Religion		
Muslim	1938 (95.3)	1073 (96.1)
Christian	82 (4.0)	37 (3.3)
Other	13 (0.6)	6 (0.5)

Race		
Arabs	1625 (79.9)	912 (81.6)
Non-Arabs	409 (20.1)	205 (18.4)

Education		
Illiterate	425 (20.9)	257 (23.0)
≤ 9 years	1131 (55.5)	578 (51.7)
> 9 years	482 (23.7)	283 (25.3)

	mean ± SD	mean ± SD

Total number of people in the household	6.5 ± 3.3	6.7 ± 3.2
Adults	3.3 ± 1.8	3.5 ± 1.9
Children	3.2 ± 2.5	3.2 ± 2.5

Household density (household/rooms)	2.2 ± 1.3	2.2 ± 1.3

Total SES score	4.3 ± 2.0	4.0 ± 2.0

**Table 2:** Various indicators of exposure to ETS among adults non-smokers (n = 1118) in Aleppo, Syria.

	Non-smokers with CO≤10 ppm n (%)
Spouse's smoking (cigarettes and waterpipe)	351 (43.7)*
	
Parental smoking	
None	374 (33.5)
Father	591 (52.9)
Mother	36 (3.2)
Both	117 (10.5)
	
Number of household smokers	
Cigarettes ≥ 1 smoker	543 (48.6)
Waterpipe ≥ 1 smoker	47 (4.2)
Both ≥ 1 smoker	108 (9.7)
	
Past year regular exposure to other's smoke	769 (68.8)
	
Past week sensory irritation from ETS exposure	
Sometimes	188 (16.8)
Many times	103 (9.2)
Past week hours spent daily with smokers	
At home	
≤ 1 hour/day	651 (58.2)
>1 hour/day	467 (41.8)
At other places	
≤ 1 hour/day	795 (71.1)
>1 hour/day	323 (28.9)
	
Exposure to smoking at workplace	
Yes/well ventilated	205 (58.9)*
Yes/poor ventilated	32 (9.2)*
	
Average cigarettes smoked daily in the house	
1–10 cig/day	438 (39.2)
> 10 cig/day	281 (25.1)
	
Average waterpipes smoked daily in the house	
1–2 waterpipe/day	33 (3.0)
> 2 waterpipe/day	8 (0.7)
	
House policy regarding smoking	
Smoking is not allowed at all	40 (3.6)
Smoking is allowed for only few guests	137 (12.3)
Smoking is allowed only in special places	106 (9.5)
Smoking is not restricted at all	812 (72.6)
Differs between cigarettes and waterpipes	23 (2.1)

* Calculated from the number of non-smokers who are currently married (n = 803), or employed (n = 348)

### Outcomes

Past year recurrent cough and recurrent shortness of breath were defined as having 3 or more recognizable episodes of these symptoms. Those reporting recurrent cough or shortness of breath were asked to select, from a provided list, one or more options that best described their symptoms (Table 3). The main asthma symptom (past year wheezing/whistling in the chest) and asthma diagnosis were inquired about from all participants, while other asthma symptoms (recurrent cough accompanied by wheezing, recurrent nocturnal cough unrelated to colds, and recurrent episodic shortness of breath accompanied by wheezing) were inquired about among those reporting recurrent cough or shortness of breath. Items related to physician-diagnosed conditions included ever having a diagnosis of (asthma, chronic bronchitis, or emphysema), or the occurrence of a diagnosed condition (sinusitis, acute bronchitis, pneumonia) during the past year. Hay fever was defined conservatively based on positive responses to two questions about past year nasal allergic symptoms (episodes of sneezing, runny or blocked nose when not experiencing a cold), and the co-occurrence of itchy and watery eyes [38]. Severity of respiratory complaints was based on the number of wheezing/whistling episodes for asthma (≤ 12 and > 12), reporting more than one episode of sinusitis or acute lower respiratory tract infection, and medical care (medication use, hospital or clinic visits) for respiratory problems (Table 4). Medication or health facility use because of respiratory problems was broken down further by condition (asthma, pneumonia, bronchitis, etc.), but because none of these outcomes were associated with exposure to ETS in our study we used only the parent general question.

**Table 3:** Relation between different levels of exposure to ETS and respiratory symptoms/diagnosis among adult non-smokers in Aleppo-Syria (n = 1118)

	ETS score*		
*Self-reported respiratory symptoms/diagnoses*	middle	high	*P *Dose-response
**General respiratory symptoms**			
Past year recurrent cough (≥ 3 recognizable episodes)	1.3 (0.8–1.9)	1.9 (1.2–2.9)	0.004
Past year recurrent shortness of breath (≥ 3 recognizable episodes)	1.6 (1.1–2.3)	1.7 (1.1–2.6)	0.001
Past year recurrent exertional shortness of breath that disappears after rest	1.8 (1.2–2.7)	2.0 (1.3–3.2)	<0.001
Past year recurrent shortness of breath almost all the time	3.0 (1.3–6.8)	2.6 (1.1–6.3)	0.02
**Symptoms/diagnosis suggestive of asthma**			
Past year wheezing/whistling in the chest	1.4 (0.8–2.2)	1.7 (1.0–2.8)	0.05
Past year recurrent episodic dry cough accompanied by wheezing/whistling	1.9 (1.0–3.7)	1.9 (0.9–3.9)	0.05
Past year recurrent nocturnal cough, not related to colds, that wakes the subject up	1.2 (0.7–1.9)	1.9 (1.1–3.2)	0.02
Past year recurrent episodic shortness of breath accompanied by wheezing	2.2 (1.1–4.4)	1.6 (0.7–3.7)	0.06
Ever diagnosed asthma	1.8 (1.2–2.8)	1.4 (0.9–2.4)	0.1
Hay fever (nasal allergy symptoms with eye itching and watering)	0.9 (0.6–1.3)	1.5 (0.9–2.3)	0.01
**Symptoms/diagnosis suggestive of chronic bronchitis**			
Productive cough that lasts most of the winter	1.2 (0.7–2.2)	1.6 (0.8–2.9)	0.2
Recurrent shortness of breath accompanied by cough and phlegm	2.2 (1.1–4.7)	2.5 (1.1–5.6)	0.02
Ever diagnosed chronic bronchitis/emphysema	1.1 (0.5–2.2)	1.2 (0.5–2.7)	0.6
**Symptoms/diagnosis suggestive of respiratory infection**			
Past year recurrent cough accompanying upper respiratory infections (cold, flue)	1.0 (0.7–1.6)	1.5 (0.9–2.4)	0.1
Past year recurrent cough with bloody phlegm	1.1 (0.5–2.6)	1.4 (0.6–3.4)	0.7
Past year sinusitis	1.0 (0.6–1.7)	1.7 (1.0–2.9)	0.09
Past year diagnosed acute lower respiratory infection (bronchitis, pneumonia)	1.3 (0.7–2.3)	1.9 (1.1–3.6)	0.03

* Odds ratio and 95% confidence interval for the relation between ETS score tertiles (lower being referent) and respiratory symptoms/diagnosis according to multivariate logistic regression adjusted for age, gender, SES score, hay fever, parental allergy, pack-years (for ex-daily smokers), occupational exposure to respirable pollutants other than ETS

**Table 4:** Relation between different levels of exposure to ETS and severity of respiratory problems of adult non-smokers in Aleppo-Syria

	ETS score*		
*Severity of respiratory problems*	middle	high	*P *Dose-response
Number of wheezing attacks in the past year (≤12 vs. >12)	0.6 (0.2–2.2)	1.2 (0.3–4.5)	0.6
Number of episodes of sinusitis (once vs. more than once)	0.5 (0.2–1.4)	1.2 (0.4–4.1)	0.3
Number of episodes of acute lower respiratory tract infection (once vs. more than once)	1.4 (0.4–4.6)	0.7 (0.2–2.3)	0.3
Past year doctor's or hospital visit because of respiratory problems	1.4 (0.9–2.1)	1.2 (0.7–2.0)	0.4
Past month medication use for respiratory problems	1.0 (0.5–1.7)	1.0 (0.5–1.9)	0.7

* Odds ratio and 95% confidence interval for the relation between ETS score tertiles (lower being referent) and respiratory symptoms/diagnosis according to multivariate logistic regression adjusted for age, gender, SES score, hay fever, parental allergy, pack-years (for ex-daily smokers), occupational exposure to respirable pollutants other than ETS

Forced Expiratory Volume in the 1^st ^second (FEV_1_) and Forced Vital Capacity (FVC) were measured for all participants according to standard guidelines [39]. We used hand-held spirometer (Micro-plus, Micro Medical, Rochester, UK), which have been shown to have good precision and reproducibility [40]. We used newly calibrated spirometers and tested them weekly by team members with known lung functions (allowing for no more than 5% variation between different spirometers). Multiple maneuvers were performed until three satisfactory ones were recorded. The best effort that did not exceed the next best by more than 5% was included in the analysis [41] (Table 5).

**Table 5:** Relation between exposure to ETS and lung functions among men and women non-smokers (n = 623) in Aleppo-Syria

	ETS score		
	middle	high	*P *Dose-response
Men			
FEV_1 _(ml)	-46.8 (-215.4 to 121.7)	34.3 (-140.5 to 209.1)	0.3
FVC (ml)	63.9 (-167.5 to 295.3)	147.9 (-89.1 to 385.0)	0.5
FEV_1_/FVC (%)	-1.2 (-3.7 to 1.6)	-1.0 (-3.5 to 1.6)	0.5
			

Women			
FEV1 (ml)	-87.8 (-164.8 to -10.7)	-58.7 (-136.5 to 19.1)	0.3
FVC (ml)	3.1 (-103.8 to 110.1)	-72.2 (-182.4 to 38.1)	0.2
FEV_1_/FVC (%)	-2.0 (-3.8 to -0.3)	-1.0 (-2.9 to 0.8)	0.1

* Unstandardized linear regression coefficient and 95% confidence interval for the relation between ETS score tertiles (lower being referent) and lung function tests adjusted for age, BMI, SES score, hay fever, pack years (for ex-cigarette smokers), occupational exposure, parental allergy, and interaction terms of age with height and age with weight

### Analysis

Descriptive statistics were calculated for the overall study population and for measures of ETS exposure among non-smokers (Tables 1,2). Composite scores for socioeconomic status (SES score) and self-reported ETS exposure indices were constructed for the analysis (as illustrated in the additional file, Appendix 1). Spearman correlation coefficients were calculated to assess the relation between FEV_1_, FVC, FEV_1_/FVC and ETS score. Logistic regression was used to estimate the odds ratio (OR) and the 95% confidence interval for the relation between ETS score and respiratory symptoms adjusting for age, sex, SES score, parental allergy, occupational exposure to other respiratory pollutants, and pack years (for ex-daily smokers). Linear regression analysis was used to assess the relationship between ETS score and lung function (FEV_1_, FVC, and FEV_1_/FVC) adjusting for age, BMI, SES score, and occupational exposure to other respiratory irritants, as well as interaction terms of age with height and weight. This analysis was performed separately for men and women, since lung development and response to ETS has been shown to differ by gender [26,28]. In both multivariate models (logistic, linear), ETS score was first entered as a categorical variable (low; bottom tertile, middle; middle tertile, high; top tertile) for the calculation of OR for different gradients of exposure, then as continuous variable for the calculation of *p *for dose-response relationship. Because of the concern that ex-smokers may avoid ETS exposure and have respiratory problems (giving us a group with potentially most respiratory problems but least exposure), we repeated the analysis including only never smokers, but this did not affect the results considerably (analysis not shown). All analyses were done by SPSS 11.

## Results

From a total of 2038 valid survey responses (45.2% men, mean age 35.3 ± 12.1, response rate 86%), 1118 (27.1% men) satisfied the inclusion criteria for the exposure-symptoms analysis (Table 1), and 623 (30% men) for the exposure-lung functions analysis (Table 5). According to ETS score (mean ± SD 8.8 ± 3.6, median 9), the vast majority of non-smokers in our population were exposed to ETS, whereby only 3.6% had levels ≤ 2 and 21.1% had levels ≤ 5 (Table 2).

Logistic regression analysis of the relation between ETS score and self-reported symptoms/diagnosis generally shows a dose-response association with symptoms and diagnosed acute lower respiratory tract infection (acute bronchitis, pneumonia). General respiratory symptoms associated with exposure to ETS were past year recurrent cough (ORs for comparison between middle, high, with the low exposure group were 1.3 and 1.9, respectively, with *p *for dose response *0.004*), past year recurrent shortness of breath (ORs 1.6 and 1.7, *p = 0.001*), past year recurrent exertional shortness of breath that disappears after rest (ORs 1.8 and 2, *p < 0.001*), past year recurrent shortness of breath almost all of the time (ORs 3 and 2.6, *p = 0.02*). Additionally, several symptoms suggestive of asthma/allergy were related to ETS exposure, including past year recurrent wheezing/whistling in the chest (ORs 1.4 and 1.7, *p = 0.05*), past year recurrent episodic dry cough accompanied by wheezing (ORs 1.9 and 1.9, *p = 0.05*), past year recurrent episodic shortness of breath accompanied by wheezing (ORs 2.2 and 1.6, *p = 0.06*), past year recurrent nocturnal cough not related to cold that wakes the subject up (ORs 1.2 and 1.9, *p = 0.02*), and past year hay fever symptoms (ORs 0.9 and 1.5, *p = 0.01*). Among symptoms suggestive of chronic bronchitis, ETS exposure was associated with recurrent shortness of breath accompanied by cough and phlegm (ORs 2.2 and 2.5, *p = 0.02*). And finally, past year episodes of acute lower respiratory infection (bronchitis, pneumonia) were associated with exposure to ETS (OR 1.3 and 1.9, *p = 0.03*) (Table 3).

In contrast, exposure to ETS was not related to severity of respiratory complaints judged by the number of episodes (wheezing, sinusitis, acute bronchitis, pneumonia), medical care utilization for respiratory problems in general (Table 4), as well as medical care utilization for a specific problem (e.g. asthma, pneumonia, analysis not shown).

In the univariate analysis, there was a weak inverse correlation between ETS score and FEV_1 _(coefficient -0.1, *p < 0.001*), FVC (-0.1, *p = 0.002*), and %FEV1/FVC (-0.6, *p = 0.1*). Linear regression analysis between ETS exposure score and lung functions showed significant inverse associations with indices of airflow limitation (FEV_1 _and FEV_1_/FVC) only in women. Women in the middle category of ETS exposure had on average 88 ml deficit in FEV_1 _and 2% in FEV_1_/FVC in comparison to those in the low exposure category. This association did not show a dose-response relationship (Table 5).

## Discussion

This study shows that exposure to ETS is universal among non-smoking adults in Aleppo-Syria. This exposure is associated with respiratory complaints of both infectious and non-infectious etiology in a dose-response fashion, suggesting a causal relationship. Unlike data from developed countries, however, exposure to ETS was not related to increased severity of asthma or other respiratory conditions judged by symptoms frequency and medical care utilization because of respiratory problems. ETS exposure was associated with decrements in lung functions suggestive of airflow limitation (FEV_1 _and FEV_1_/FVC) among women only.

Error and bias in ascertainment are always a concern in cross-sectional studies, due to imprecise or differential recall. We tried to minimize such problems by not highlighting tobacco or ETS exposure when introducing the study to participants [42], by using symptoms/diagnosis descriptors that are native to the target population, and by assessing multiple indices of both exposures and outcomes. We have some indicators that such bias was limited, including lack of associations between a diagnosis of asthma or chronic bronchitis and ETS score. Remarkably, recurrent cough with bloody phlegm, one of the potentially most startling respiratory symptoms, was not associated with ETS exposure in this study, giving further support of minimal recall bias. Our reliance on self-reported exposure is also a potential limitation, but studies have repeatedly shown that self-report is a valid measure of ETS exposure that correlates with other objective markers such as cotinine [43-46]. Understandably, the composite ETS score is a crude quantitative measure of ETS exposure. We opted for its use to incorporate various sources of information about exposure to ETS in order to differentiate between meaningful gradients of exposure for the purpose of the analysis. On the other hand, the diversity of information relevant to ETS exposure considered in this study, in addition to the verification of non-smoking status by breath CO measurement, and the use of multiple subjective and objective outcomes, helped delineate the exposed group and conduct a robust analysis.

The widespread exposure to ETS among adults in Aleppo, suggests that it is rather hard to avoid such exposure in this environment. The vast majority of non-smokers in Aleppo are exposed to ETS both at home and outside. This exposure is sufficiently intense to cause sensory (eye and nose) irritation for a quarter of nonsmokers. In comparison, less than a quarter of non-smokers in a national sample of 43,732 adults in the US report exposure to ETS [47]. Interestingly, such spread of exposure is occurring in the face of enacted laws banning smoking in public buildings, worksites, and transportation in Syria since the nineties [48]. Our results indicate that these laws are not enforced, as about two thirds of working non-smokers report exposure to others' smoke at work. Accordingly, these results should provide solid ground for public health advocates and authorities to push for the application of policies and measures to protect non-smokers from this hazardous exposure. Although not in the realm of laws and regulations, the widespread liberal attitude towards smoking in the house suggests a general lack of awareness, or dismissal, of the health damaging effects of ETS exposure to household members. Remarkably, about three quarters of studied households do not restrict indoor smoking whatsoever, and only a small minority (3.6%) has total restriction. This shows that in societies where smoking is rather a norm, it becomes hard to employ smoking restrictions even in one's own house. Increasing public awareness of this health hazard is thus an area where public health advocacy can make a difference.

In general, this study shows stronger and dose-dependent relationships of ETS exposure with general respiratory symptoms (i.e. recurrent cough or shortness of breath) than with symptoms characteristic of specific respiratory problems (asthma, bronchitis). Arguably, general symptoms are more easily identifiable as well as shared among many respiratory problems. The magnitude of difference in self-reported asthma symptoms according to exposure level is generally in the range of a twofold increase. This effect magnitude is similar to that reported in adults from 16 European countries (European Community Respiratory Health Survey, ECRHS), but lower than that reported from the Swiss Study on Air Pollution and Lung Diseases in Adults (SAPALDIA) [49,50]. Similar to studies from developed countries, we found a dose-response relationship between exposure to ETS and respiratory symptoms, implying a causal relationship [28,49-52]. On the other hand, unlike data from European nations (ECRHS), we found a 50% increase in hay fever symptoms for the high exposure group compared to those with low exposure. We defined hay fever according to symptom report, however, while the ECRHS inquired about suffering from allergic rhinitis or hay fever [49]. Also, among symptoms indicative of chronic bronchitis, only recurrent shortness of breath with cough and phlegm was associated with ETS exposure in our study, while in the ECRHS ETS exposure during childhood was associated with increased reporting of recurrent cough and phlegm in adulthood [28]. Since cough and phlegm are common symptoms of infectious respiratory problems, likely to be widespread in our population due to overcrowding and poor housing conditions, such symptoms can be non-specific indicators of chronic bronchitis in our setting, while shortness of breath can be more specific marker of this condition.

Level of exposure to ETS in our population was not associated with severity of asthma, sinusitis, or lower respiratory tract infection. In contrast, in a study of 349 adults with asthma in the US, Eisner and colleagues found that exposure to ETS at baseline was associated with more symptom severity and emergency/hospital admissions because of asthma at 18 month followup [53]. Medical care utilization for respiratory problems is likely to be an inadequate indicator of severity in a low-income country such as Syria, where the lack of medical insurance and limited public health services render seeking private health care the last resort for most adults in this country. On the other hand, as it will be discussed later, the lack of a comparison group of non-exposed individuals may have contributed to the absence of association between ETS exposure and symptoms severity in this study.

Studies of the relation between exposure to ETS and lung function have generally shown a detrimental effect of such exposure. This effect, however, was not consistent and of low magnitude generally (50–100 ml) [3]. For example, in a population-based sample of adults from the NHANES III survey in the US, exposure to ETS was associated with decreased lung functions (FEV_1_, FVC, FEV_1_/FVC) in women but not men [26]. Data from the ECRHS involving 18,922 adults from 17 European countries show that exposure to parental smoking in childhood was associated with impaired lung function [28]. The effect on lung function differed, however, according to participant's gender, parental smoking (mother, father, both), and period of exposure (during pregnancy, childhood) [28]. Our study shows that exposure to ETS is associated with decreased lung function indicative of airflow limitation (FEV_1_, and FEV_1_/FVC) in female but not male non-smokers. The reduction for the middle compared to low exposure category was in the magnitude of 88 ml for FEV_1 _and 2% for FEV_1_/FVC. Although other studies have reported similar gender-specific vulnerability of women [26], it is important to emphasize the small number of male non-smokers in our sample (less than one third). We also could not elicit a dose-response in the relation between ETS exposure and airflow limitation among women, although ETS score was weakly correlated to FVE_1 _in the univariate analysis. It is possible that we are dealing in our setting with levels of exposure that exceed those occurring in western societies. Indeed, because of the low magnitude of the effect of ETS exposure on lung function, studies have relied on comparisons of exposed vs. non-exposed individuals to assess this relationship [49,53-55]. In our sample, however, we had very few subjects with no or little exposure, which could have reduced the sensitivity of our analysis. Another possibility is that the relatively crude measure of ETS (ETS score) we used may better differentiate between gradients of exposure at its lower stratum than higher.

## Conclusions

This study shows that exposure to ETS is rampant among adult non-smokers in Syria, where it is hard to escape it due to a high prevalence of smoking, household over-crowding, and lack of smoking restrictions. This exposure is leading to increased respiratory symptoms/disease of both infectious and non infectious etiologies, and is likely to have deleterious effects on respiratory function among women. In addition, the dose-response association found between exposure to ETS and respiratory symptoms point towards causal relationship. These results send a clear message to health advocates and policy makers about the spread and harmful effects of exposure to ETS in Syria and on the importance of collective efforts to educate both the public and authorities about this major health hazard and ways to effectively protect non-smokers from it. In addition to giving further support to the health hazards of ETS exposure, this study can provide guidance for future research on this issue in other developing countries.

## List of abbreviations

• AC- air condition

• AHS- Aleppo Household Survey

• ECRHS- European Community Respiratory Health Survey

• ETS- environmental tobacco smoke

• FEV_1_- forced expiratory volume in the 1^st ^second

• FVC- forced vital capacity

• NHANES III- third National Health and Nutrition Examination Survey

• OR- odds ratio

• PC- personal computer.

• ppm- part per million

• PPS- probability proportionate to size

• SAPALDIA Swiss Study on Air Pollution and Lung Diseases in Adults

• SD-standard deviation

• TV- television

## Authors' contribution

W Maziak, designed the study, conducted the analysis and wrote the 1^st ^draft of the manuscript. KD Ward, T Eissenberg, participated in the study design and co-authored the manuscript. S Rastam and F Mzayek participated in the data management, analysis, and co-authored the manuscript.

## Supplementary Material

Additional File 1 Appendix 1 : Composite scores for SES and ETS used in the study with the total score categorized around tertile cut off points. Click here for file
