# Supplementary material for: Extent of exposure to environmental tobacco smoke (ETS) and its dose-response relation to respiratory health among adults
Source: Respir Res. 2005 Feb 8;6(1):13. doi: 10.1186/1465-9921-6-13 (PMC549073; doi:10.1186/1465-9921-6-13)
Supplement: Additional File 1 — Appendix 1 : Composite scores for SES and ETS used in the study with the total score categorized around tertile cut off points. [file 1465-9921-6-13-S1.doc]

Appendix 1: Composite scores for SES and ETS used in the study with the total score categorized around tertile cut off points.

|  | **Low** (value 0) | **Middle** (value 1) | **High** (value 2) |
| --- | --- | --- | --- |
| **Socioeconomic status (maximum 12)**    Education    Employment    Items ownership (phone, mobile phone, PC, AC, private car, TV, satellite dish)    Household members with paid job    Self reported monthly income    Density index  **Total (tertiles)** | illiterate  unemployed, student  < 2  0  < 10,000 SL  > 2.3  **< 3** | < 9 years  employed (manual, private, government), retired  3-4  1  10,000-20,000  1.5-2.3  **4-5** | > 9 years  employer, private business (including engineers, lawyers, etc.)  > 4 or private car  > 1  > 20,000  > 1.5  **> 5** |
| **ETS exposure score (maximum 22)**  Spouse’s smoking (cigarettes and waterpipe)    Parental smoking    Number of household smokers  Cigarettes  Waterpipe  Past year regular exposure to other’s smoke  Past week sensory irritation from ETS exposure  Past week hours spent daily with smokers (in the house and outside)  Exposure to smoking at workplace  Average cigarettes smoked daily in the house    Average waterpipes smoked daily in the house  House policy regarding smoking  **Total (tertiles)** | no  no  no  no  no  no  no  no  0  0  smoking not allowed  **< 7** | < 10 pack yrs and/or waterpipe for < 5 yrs  one parent for less than 10 years  < 1  < 1  -  sometimes  < 2  yes/well ventilated  < 10 cigarettes  1-2 waterpipes  allowed for few gusts  allowed in special places  **8-11** | > 10 pack yrs and/or waterpipe for > 5 yrs  both parents or one parent for more than 10 years  > 1  > 1  yes  many times  > 2  yes/poorly ventilated  > 10 cigarettes  > 2 waterpipes  no restrictions/restrictions on one form of smoking  **> 11** |
